# 2-[4-Chloro-3-(4-ethoxy­benz­yl)phen­yl]-1,3-dithiane

**DOI:** 10.1107/S1600536810017393

**Published:** 2010-05-19

**Authors:** Brian Samas, Cathy Préville, Benjamin A Thuma, Vincent Mascitti

**Affiliations:** a Pharmaceutical Sciences, Pfizer Global Research & Development, Groton Laboratories, Eastern Point Rd, Groton, CT 06340, USA; b CVMED Medicinal Chemistry, Pfizer Global Research & Development, Groton Laboratories, Eastern Point Rd, Groton, CT 06340, USA

## Abstract

In the title compound, C_19_H_21_ClOS_2_, the dithiane ring adopts a chair conformation. The dihedral angle between the benzene rings is 87.88 (4)°. In the crystal, inversion dimmers linked by pairs of C—H⋯O inter­actions occur.

## Related literature

For a related structure, see: Fun *et al.* (2009 ▶). For diaryl­methane motifs, see: Xu *et al.* (2009 ▶).
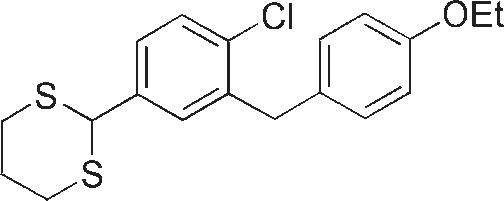


## Experimental

### 

#### Crystal data

C_19_H_21_ClOS_2_
*M* *_r_* = 364.93
Monoclinic, 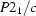

*a* = 15.8214 (3) Å
*b* = 12.2444 (2) Å
*c* = 9.4191 (2) Å
β = 100.715 (1)°
*V* = 1792.89 (6) Å^3^
*Z* = 4
Cu *K*α radiation
μ = 4.06 mm^−1^
*T* = 100 K
0.19 × 0.13 × 0.03 mm

#### Data collection

Bruker APEXII CCD diffractometer
Absorption correction: multi-scan (*SADABS*; Sheldrick, 2002 ▶) *T*_min_ = 0.513, *T*_max_ = 0.888
13885 measured reflections
3257 independent reflections
3040 reflections with *I* > 2σ(*I*)
*R*_int_ = 0.025

#### Refinement

*R*[*F* ^2^ > 2σ(*F* ^2^)] = 0.035
*wR*(*F* ^2^) = 0.095
*S* = 1.06
3257 reflections
209 parameters
H-atom parameters constrained
Δρ_max_ = 0.41 e Å^−3^
Δρ_min_ = −0.22 e Å^−3^

### 

Data collection: *APEX2* (Bruker, 2006 ▶); cell refinement: *SAINT-Plus* (Bruker, 2006 ▶); data reduction: *SAINT-Plus*; program(s) used to solve structure: *SHELXS97* (Sheldrick, 2008 ▶); program(s) used to refine structure: *SHELXL97* (Sheldrick, 2008 ▶); molecular graphics: *SHELXTL* (Sheldrick, 2008 ▶) and *Mercury* (Macrae *et al.*, 2006 ▶); software used to prepare material for publication: *SHELXTL*.

## Supplementary Material

Crystal structure: contains datablocks I, global. DOI: 10.1107/S1600536810017393/ez2206sup1.cif

Structure factors: contains datablocks I. DOI: 10.1107/S1600536810017393/ez2206Isup2.hkl

Additional supplementary materials:  crystallographic information; 3D view; checkCIF report

## Figures and Tables

**Table 1:** Hydrogen-bond geometry (Å, °)

*D*—H⋯*A*	*D*—H	H⋯*A*	*D*⋯*A*	*D*—H⋯*A*
C19—H19*A*⋯O1^i^	0.97	2.40	3.367 (2)	173

Symmetry code: (i) 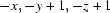.

**Fig. 1.**
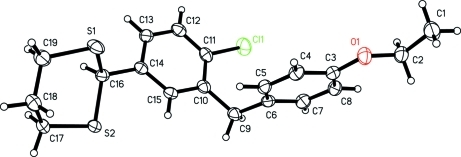
View of the constituents of (I), showing the atom-labeling scheme and displacement ellipsoids drawn at the 50% probability level. H atoms are represented by circles of arbitrary size.

**Fig. 2.**
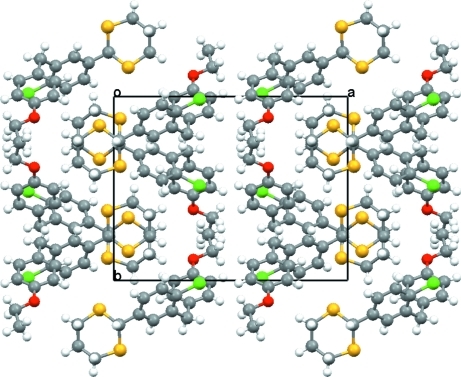
A partial packing diagram viewed along the c direction.

**Fig. 3.**
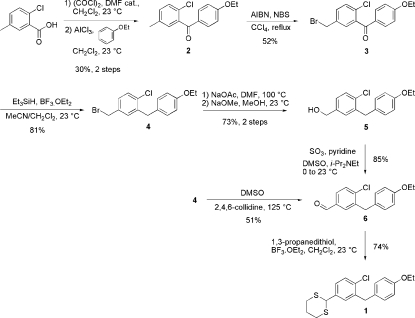
Reaction scheme.

## supplementary crystallographic information

### Comment

Diarylmethane motifs are found in some C-aryl glucoside anti-diabetic agents prototypes. Gaining understanding of these motifs is of interest to the pharmaceutical industry (Xu *et al.*, 2009). The asymmetric unit contains one molecule of the title compound. There are no classic hydrogen bonds in the structure. A weak C-H···O interaction pairs the molecules in a head-to-tail fashion. Crystal packing is controlled by van der Waals forces and π–π stacking interactions between both rings.

The separate π–π interactions, rings C3-C8 and C10-C15, are almost normal to each other at 87.88 (4)° and stabilize the structure. The rings are configured in a parallel-displaced configuration, the distance from closest contacts (C5-C15) is 3.599 (2) Å and measured from centroid-to-centroid these rings are separated by 4.8391 (1) Å.

The dithiane ring is in the expected chair conformation with a slight twisting in reference to the benzene ring, to avoid steric repulsion. The saturated ring exhibits a slight outward distortion from the C—S, due to the longer C—S bond. From the dithiane ring the benzene ring is in the expected equatorial position. These features are all common to dithiane crystal structures (Fun *et al.*, 2009). Viewed down the c axis, the dithiane rings inter-leave (Figure 2).

### Experimental

A synthesis of compound 1 is described on Fig. 3. Friedel Craft acylation of ethoxy benzene with 2-chloro-5-methyl benzoyl chloride in dichloromethane in presence of aluminum trichloride produced intermediate 2 in 30% yield. Radical bromination of 2 under classical conditions (AIBN, NBS, CCl_4_) gave 3 (52% yield) which was subsequently treated with triethylsilane and boron trifluoride diethyl etherate to provide 4 in 81% yield. Nucleophilic displacement with sodium acetate followed by deacetylation (NaOMe/MeOH) led to intermediate 5 in 73% yield over 2 steps. Parikh-Doering oxidation produced the desired aldehyde intermediate 6 in 85% yield. Alternatively, Kornblum oxidation of 4 using 2,4,6-collidine in presence of dimethylsulfoxide gave 6 in 51% yield. Treatment of 6 with 1,3-propanedithiol in the presence of boron trifluoride diethyl etherate cleanly produced dithiane 1 in 74% yield. 1 was dissolved in ethyl acetate and allowed to evaporate over twelve hours to form X-ray diffraction quality crystals. Further details of the synthesis are included in the Experimental Special Details section.

### Refinement

All H atoms were positioned geometrically and refined using a riding model, with C—H = 0.93 Å for aryl, 0.97 Å for methylene, and 0.96 Å for methyl H atoms. *U*_iso_(H) = 1.2*U*_eq_(C) for aryl and methylene H atoms, and 1.5*U*_eq_(C) for methyl H atoms.

### Crystal data

**Table:**

C_19_H_21_ClOS_2_	*F*(000) = 768
*M**_r_* = 364.93	*D*_x_ = 1.352 Mg m^−^^3^
Monoclinic, *P*2_1_/*c*	Cu *K*α radiation, λ = 1.54178 Å
Hall symbol: -P 2ybc	Cell parameters from 9696 reflections
*a* = 15.8214 (3) Å	θ = 2.8–68.7°
*b* = 12.2444 (2) Å	µ = 4.06 mm^−^^1^
*c* = 9.4191 (2) Å	*T* = 100 K
β = 100.715 (1)°	Block, colourless
*V* = 1792.89 (6) Å^3^	0.19 × 0.13 × 0.03 mm
*Z* = 4	

### Data collection

**Table:**

Bruker APEXII CCD diffractometer	3257 independent reflections
Radiation source: rotating anode	3040 reflections with *I* > 2σ(*I*)
Montel Multilayer optics	*R*_int_ = 0.025
φ and ω scans	θ_max_ = 69.3°, θ_min_ = 2.8°
Absorption correction: multi-scan (*SADABS*; Sheldrick, 2002)	*h* = −19→19
*T*_min_ = 0.513, *T*_max_ = 0.888	*k* = −13→14
13885 measured reflections	*l* = −7→11

### Refinement

**Table:**

Refinement on *F*^2^	Primary atom site location: structure-invariant direct methods
Least-squares matrix: full	Secondary atom site location: difference Fourier map
*R*[*F*^2^ > 2σ(*F*^2^)] = 0.035	Hydrogen site location: inferred from neighbouring sites
*wR*(*F*^2^) = 0.095	H-atom parameters constrained
*S* = 1.06	*w* = 1/[σ^2^(*F*_o_^2^) + (0.0597*P*)^2^ + 0.711*P*] where *P* = (*F*_o_^2^ + 2*F*_c_^2^)/3
3257 reflections	(Δ/σ)_max_ = 0.001
209 parameters	Δρ_max_ = 0.41 e Å^−^^3^
0 restraints	Δρ_min_ = −0.22 e Å^−^^3^
0 constraints	

### Special details

**Table:**

Experimental. (2-Chloro-5-methyl-phenyl)-(4-ethoxy-phenyl)-methanone (2):To a solution of 2-chloro-5-methyl-benzoic acid (1.0 g, 5.86 mmol) in dichloromethane (12 ml) was added at room temperature (~23°C) oxalyl chloride (0.54 ml, 6.2 mmol) followed by the dropwise addition of *N*,*N*-dimethylformamide (0.1 ml). The resulting solution was allowed to stir overnight at room temperature before being concentrated under reduced pressure to produce crude 2-chloro-5-methylbenzoyl chloride which was used in the next step without further purification. The crude 2-chloro-5-methylbenzoyl chloride (1.15 g, 5.86 mmol) and ethoxybenzene (0.75 g, 6.14 mmol) were dissolved in 10 ml of dichloromethane and the resulting solution was cooled to 0°C. Aluminum trichloride (0.85 g, 6.38 mmol) was added and the reaction mixture was allowed to stir at 0°C for 4 hours before being allowed to warm to room temperature overnight (~16 hours). The reaction was quenched by pouring the solution over ice. The mixture was diluted with water and extracted three times with dichloromethane. The combined organic layers were successively washed with aqueous 1 N hydrochloric acid solution, aqueous 1 N sodium hydroxide solution, brine, dried over sodium sulfate, filtered and concentrated under reduced pressure. The crude material was chromatographed using an ISCO automated chromatography unit (40 g silica gel column) and eluting with a gradient of 0-40% ethyl acetate in heptane yielding 650 mg (30% yield) of an oil that solidified upon standing. MS (LCMS) 274.9 (M+H^+^; positive mode). HRMS calculated for C_16_H_16_ClO_2_ (M+H^+^) 275.0833 found 275.0832. ^1^H NMR (400 MHz, CHLOROFORM-d) δ ppm 1.43 (t, J=7.0 Hz, 3 H), 2.34 (s, 3 H), 4.09 (q, J=7.0 Hz, 2 H), 6.90 (d, J=8.8 Hz, 2 H), 7.14 (s, 1 H), 7.17 - 7.22 (m, 1 H), 7.30 (d, J=8.2 Hz, 1 H), 7.76 (d, J=8.8 Hz, 2 H); ^13^C NMR (100 MHz, CHLOROFORM-d) δ ppm 194.3, 163.7, 139.0, 136.9, 132,7 (2 C), 132.5, 131.7, 129.8, 129.5, 128.4, 114.4 (2 C), 64.0, 21.0, 14.8.(5-Bromomethyl-2-chloro-phenyl)-(4-ethoxy-phenyl)-methanone (3):To a solution of (2-chloro-5-methyl-phenyl)-(4-ethoxy-phenyl)-methanone (2; 650 mg, 2.37 mmol) and *N*-bromosuccinimide (465 mg, 2.61 mmol) in carbon tetrachloride (8 ml) was added 2,2'-azobisisobutyronitrile (AIBN, 8 mg, 0.047 mmol) and the reaction mixture was heated to reflux for 26 hours under nitrogen. The reaction was cooled to room temperature, quenched with water (50 ml) and the mixture diluted with dichloromethane (25 ml). The layers were separated and the aqueous layer was extracted two additional times with dichloromethane (25 ml). The combined organic layers were washed with brine, dried over magnesium sulfate, filtered through a pad of celite, and concentrated under reduced pressure. The crude material was chromatographed with an ISCO automated chromatography unit (40 g silica gel column) eluting with a gradient of 0-20% ethyl acetate in heptane to produce 434 mg (52% yield) of the desired product as a white solid. HRMS calculated for C_16_H_15_BrClO_2_ (M+H^+^) 352.9938 found 352.9939. ^1^H NMR (400 MHz, CHLOROFORM-d) δ ppm 1.43 (t, J=7.0 Hz, 3 H), 4.10 (q, J=6.9 Hz, 2 H), 4.45 (s, 2 H), 6.91 (d, J=8.8 Hz, 2 H), 7.36 (s, 1 H), 7.37 - 7.40 (m, 2 H), 7.76 (d, J=9.0 Hz, 2 H). ^13^C NMR (100 MHz, CHLOROFORM-d) δ ppm 193.3, 163.3, 139.6, 136.9 132.7 (2 C), 131.5, 131.3, 130.6, 129.5, 129.1, 114.6 (2 C), 64.1, 31.8, 14.8.4-Bromomethyl-1-chloro-2-(4-ethoxy-benzyl)-benzene (4):To a solution of (5-bromomethyl-2-chloro-phenyl)-(4-ethoxy-phenyl)-methanone (3; 400 mg, 1.13 mmol) dissolved in dichloromethane (2 ml) and acetonitrile (4 ml) cooled to 0°C was added triethylsilane (0.7 mL, 4 mmol). Boron trifluoride diethyl ether complex (0.34 ml, 2.71 mmol) was added dropwise to the stirring solution and the reaction mixture was allowed to warm to room temperature overnight. The reaction was quenched by addition of aqueous 1 N sodium hydroxide (30 ml) and the resulting biphasic mixture was extracted three times with ethyl acetate (30 ml). The combined organic layers were successively washed with aqueous 1 N sodium hydroxide solution (50 ml), brine (50 ml), dried over sodium sulfate, filtered and concentrated under reduced pressure. The crude material was chromatographed using an ISCO automated chromatography unit (12 g silica gel column) eluting with a gradient of 0-50% ethyl acetate in heptane to produce 310 mg (81% yield) of the desired product as a clear oil. HRMS calculated for C_16_H_17_BrClO (M+H^+^) 339.0145 found 339.0137. ^1^H NMR (400 MHz, CHLOROFORM-d) δ ppm 1.39 (t, J=7.0 Hz, 3 H), 4.00 (q, J=8.4 Hz, 2 H), 4.01 (s, 2 H), 4.38 (s, 2 H), 6.82 (d, J=8.6 Hz, 2 H), 7.08 (d, J=8.6 Hz, 2 H), 7.12 (d, J=2.0 Hz, 1 H), 7.17 (dd, J=8.1, 2.0 Hz, 1 H), 7.32 (d, J=8.2 Hz, 1 H). ^13^C NMR (100 MHz, CHLOROFORM-d) δ ppm 157.7, 139.8, 136.7, 134.4, 131.5, 131.1, 130.2, 130.1 (2 C), 128.4, 114.7 (2 C), 63.6, 38.4, 32.8, 15.1.[4-Chloro-3-(4-ethoxy-benzyl)-phenyl]-methanol (5):A solution of 4-bromomethyl-1-chloro-2-(4-ethoxy-benzyl)-benzene (4; 300 mg, 0.883 mmol) and sodium acetate (217 mg, 2.65 mmol) in *N*,*N*-dimethylformamide (2 ml) was heated at 100°C for 18 hours. After cooling to room temperature, the reaction was quenched with water (30 ml) and the resulting mixture was extracted with ethyl acetate (30 ml). The aqueous layer was extracted with ethyl acetate (30 ml) two additional times. The combined organic layers were washed with brine, dried over sodium sulfate, filtered and concentrated under reduced pressure. The crude material was chromatographed using an ISCO automated chromatography unit (12 g silica gel column) eluting with a gradient of 0-50% ethyl acetate in heptane yielding 237 mg (84% yield) of acetic acid 4-chloro-3-(4-ethoxy-benzyl)-benzyl ester as a clear oil. HRMS calculated for C_18_H_19_ClNaO_3_ (M+Na^+^) 341.0914 found 341.0925. ^1^H NMR (400 MHz, CHLOROFORM-d) δ ppm 1.39 (t, J=6.9 Hz, 3 H), 2.06 (s, 3 H), 3.99 (q, 2 H), 4.02 (s, 2 H), 4.99 (s, 2 H), 6.81 (d, J=8.6 Hz, 2 H), 7.08 (d, J=8.8 Hz, 2 H), 7.10 (br. s., 1 H), 7.13 (dd, J=8.2, 2.0 Hz, 1 H), 7.34 (d, J=8.0 Hz, 1 H). ^13^C NMR (100 MHz, CHLOROFORM-d) δ ppm 170.9, 157.7, 139.6, 134.9, 134.2, 131.3, 130.9 (2 C), 130.1, 129.8, 127.5, 114.7 (2 C), 67.7, 63.6, 38.5, 21.2, 15.1.To a solution of acetic acid 4-chloro-3-(4-ethoxy-benzyl)-benzyl ester (221 mg, 0.693 mmol) dissolved in methanol (8 ml) was added 25% sodium methoxide in methanol (1 ml) until pH = 12 was reached and the reaction mixture was allowed to stir at room temperature under nitrogen for 18 hours. The mixture was neutralized with the addition of Dowex Monosphere 650 C (H) cation exchange resin (the resin was washed with methanol 3 times before using) until the pH of the solution was ~7. The reaction mixture was filtered and the filtrate was concentrated under reduced pressure. The crude material was chromatographed using an ISCO automated chromatography unit (12 g silica gel column) eluting with a gradient of 0-60% ethyl acetate in heptane to produce 167 mg (87% yield) of the desired product as a clear gum. HRMS calculated for C_16_H_17_ClNaO_2_ (M+Na^+^) 299.0809 found 299.0813. ^1^H NMR (400 MHz, CHLOROFORM-d) δ ppm 1.38 (t, J=7.0 Hz, 3 H), 1.63 (t, J=6.0 Hz, 1 H), 3.94 - 4.05 (m, 2 H), 4.02 (s, 2 H), 4.59 (d, J=5.9 Hz, 2 H), 6.81 (d, J=8.6 Hz, 2 H), 7.08 (d, J=8.6 Hz, 2 H), 7.12 (s, 1 H), 7.13 - 7.17 (m, 1 H), 7.34 (d, J=8.0 Hz, 1 H). ^13^C NMR (100 MHz, CHLOROFORM-d) δ ppm 157.7, 139.8, 139.5, 133.5, 131.5, 130.1 (2 C), 129.8, 129.6, 126.3, 114.7 (2 C), 64.8, 63.6, 38.5, 15.1.4-Chloro-3-(4-ethoxy-benzyl)-benzaldehyde (6):A solution of [4-chloro-3-(4-ethoxy-benzyl)-phenyl]-methanol (5; 150 mg, 0.542 mmol), *N*,*N*-diisopropylethylamine (0.54 ml, 3.1 mmol) and dimethylsulfoxide (0.52 ml, 7.32 mmol) in dichloromethane (3 ml) was cooled to 0°C using an ice bath. Sulfur trioxide pyridine complex (150 mg, 0.94 mmol) was added portionwise and the reaction mixture was allowed to stir at 0°C for 1 hour before being warmed to room temperature and stirred at this temperature for 2 hours. The reaction was quenched with water (50 ml), the resulting mixture was diluted with dichloromethane (25 ml) and the layers were separated. The aqueous phase was extracted with dichloromethane (25 ml) two additional times and the combined organic layers were successively washed with water (25 ml), brine (25 ml), dried over magnesium sulfate, filtered through a plug of celite, and concentrated under reduced pressure. The crude material was chromatographed using an ISCO automated chromatography unit (12 g silica gel column) eluting with a gradient of 0-40% ethyl acetate in heptane yielding 127 mg (85%) of the desired product as a clear oil which solidified upon standing. HRMS calculated for C_16_H_16_ClO_2_ (M+H^+^) 275.0833 found 275.0831. ^1^H NMR (400 MHz, CHLOROFORM-d) δ ppm 1.39 (t, J=7.0 Hz, 3 H), 4.00 (q, J=7.0 Hz, 2 H), 4.08 (s, 2 H), 6.83 (d, J=8.6 Hz, 2 H), 7.09 (d, J=8.4 Hz, 2 H), 7.52 (d, J=8.2 Hz, 1 H), 7.62 (d, J=1.8 Hz, 1 H), 7.64 - 7.69 (m, 1 H), 9.89 (s, 1 H); ^13^C NMR (100 MHz, CHLOROFORM-d) δ ppm 191.4, 157.9, 141.0, 140.7, 135.2, 132.1, 130.6, 130.4, 130.1 (2 C), 128.5, 114.9 (2 C), 63.6, 38.5, 15.1.Alternative synthesis of 6:To a solution of 4-bromomethyl-1-chloro-2-(4-ethoxy-benzyl)-benzene (4; 26.6 mg, 0.078 mmol) in dimethylsulfoxide (0.3 ml) was added 2,4,6-trimethyl-pyridine (0.03 ml, 0.2 mmol) and the reaction was heated to 125°C for 30 minutes. The reaction mixture was cooled to room temperature, water (25 ml) was added and the resulting mixture was extracted three times with ethyl acetate (20 ml). The combined organic layers were successively washed with water, brine, dried over sodium sulfate, filtered and concentrated under reduced pressure. The crude material was chromatographed using an ISCO automated chromatography unit (4.0 g silica gel column) and eluting with a gradient of 0-50% ethyl acetate in heptane yielding 11 mg (51% yield) of an oil that solidified upon standing.Alternatively 6 could be prepared in an analogous way by using the appropriate starting material: To a solution of 1-bromomethyl-4-methoxybenzene (153 mg, 0.766 mmol) in 1,2-dimethoxyethane (3 ml) was added (185 mg, 0.996 mmol), 2-chloro-5-formyl-phenyl-boronic acid a solution of 2M aqueous sodium carbonate (1.5 ml) followed by the addition [1,1'-bis(diphenyl-phosphino)ferrocene]dichloro-palladium, dichloromethane (65 mg, 0.1 mmol). The reaction mixture was purged with nitrogen before being sealed and heated to 80°C in the microwave for 30 minutes. The organic layer was separated and concentrated under reduced pressure. The crude material was chromatographed using an ISCO automated chromatography unit (12.0 g silica gel column) and eluting with a gradient of 0-70% ethyl acetate in heptane yielding 17.4 mg (9% yield) of 4-chloro-3-(4-methoxy-benzyl)-benzaldehyde as an oil.2-(4-Chloro-3-(4-ethoxy-benzyl)phenyl)-1,3-dithiane (1):To a solution of 4-chloro-3-(4-ethoxy-benzyl)benzaldehyde (8.4 g, 31 mmol) in dichloromethane (113 ml) was added 1,3-propanedithiol (3.38 ml, 33.6 mmol) and the solution was stirred at room temperature for 1 hour before cooling down to 0°C. To the solution was added boron trifluoride diethyl etherate (5 ml, 39.7 mmol) and the reaction was warmed up to room temperature over 12 hours. The mixture was diluted with dichloromethane and the reaction was quenched by dropwise addition of saturated aqueous solution of sodium bicarbonate. The organic phase was successively washed with a solution of sodium hydroxide (1M), brine, dried over magnesium sulfate, filtered and concentrated. The crude product was purified by chromatography over silica gel (0% to 30% EtOAc in heptane) to give 1 as a white solid (8.25 g, 74% yield). MS (LCMS) 365.1 (M+H^+^; positive mode). HRMS calculated for C_19_H_22_ClOS_2_ (M+H^+^) 365.0795 found 365.0796. ^1^H NMR (500 MHz, CHLOROFORM-d) δ ppm 1.42 (t, J=7.0 Hz, 3 H), 1.87 - 1.99 (m, 1 H), 2.13 - 2.21 (m, 1 H), 2.86 - 2.94 (m, 2 H), 2.99 - 3.09 (m, 2 H), 3.98 - 4.07 (m, 4 H), 5.08 (s, 1H), 6.80 - 6.88 (m, 2 H), 7.07 - 7.14 (m, 2 H), 7.23 - 7.38 (m, 3 H). ^13^C NMR (100 MHz, CHLOROFORM-d) δ ppm 15.1, 25.2, 32.2 (2 C), 38.6, 50.9, 63.6, 114.7 (2 C), 127.1, 130.1, 130.2 (2 C), 130.5, 131.2, 134.3, 138.1, 139.7, 157.7.
Geometry. All esds (except the esd in the dihedral angle between two l.s. planes)are estimated using the full covariance matrix. The cell esds are takeninto account individually in the estimation of esds in distances, anglesand torsion angles; correlations between esds in cell parameters are onlyused when they are defined by crystal symmetry. An approximate (isotropic)treatment of cell esds is used for estimating esds involving l.s. planes.
Refinement. Refinement of *F*^2^ against ALL reflections. The weighted *R*-factor wR and goodness of fit *S* are based on *F*^2^, conventional *R*-factors *R* are based on *F*, with *F* set to zero for negative *F*^2^. The threshold expression of *F*^2^ > σ(*F*^2^) is used only for calculating *R*-factors(gt) etc. and is not relevant to the choice of reflections for refinement. *R*-factors based on *F*^2^ are statistically about twice as large as those based on *F*, and *R*- factors based on ALL data will be even larger.

### Fractional atomic coordinates and isotropic or equivalent isotropic displacement parameters (Å^2^)

**Table:**

	*x*	*y*	*z*	*U*_iso_*/*U*_eq_	
S1	−0.06149 (3)	0.34104 (3)	0.16942 (5)	0.03161 (14)	
S2	0.02647 (3)	0.12144 (3)	0.18334 (5)	0.02839 (13)	
Cl1	0.36610 (3)	0.48468 (4)	0.26092 (5)	0.03858 (15)	
O1	0.34010 (7)	0.61472 (10)	0.88988 (13)	0.0309 (3)	
C10	0.24904 (10)	0.33442 (13)	0.31254 (17)	0.0245 (3)	
C17	−0.08039 (10)	0.07566 (13)	0.10028 (18)	0.0273 (3)	
H17A	−0.0852	0.0772	−0.0039	0.033*	
H17B	−0.0875	0.0005	0.1284	0.033*	
C4	0.25848 (10)	0.49708 (14)	0.72116 (18)	0.0271 (4)	
H4	0.2093	0.5232	0.7506	0.033*	
C18	−0.15249 (11)	0.14349 (14)	0.14036 (19)	0.0303 (4)	
H18A	−0.1464	0.1448	0.2447	0.036*	
H18B	−0.2070	0.1090	0.1013	0.036*	
C12	0.20648 (11)	0.45554 (14)	0.10697 (18)	0.0286 (4)	
H12	0.2196	0.5135	0.0510	0.034*	
C3	0.33940 (10)	0.53507 (13)	0.78734 (17)	0.0255 (3)	
C7	0.40363 (10)	0.41546 (14)	0.63581 (19)	0.0297 (4)	
H7	0.4529	0.3877	0.6083	0.036*	
C16	0.02258 (10)	0.25970 (12)	0.11252 (17)	0.0233 (3)	
H16	0.0101	0.2553	0.0068	0.028*	
C11	0.26600 (10)	0.41954 (14)	0.22447 (18)	0.0267 (3)	
C14	0.10798 (10)	0.31682 (13)	0.15714 (17)	0.0228 (3)	
C15	0.16857 (10)	0.28457 (13)	0.27638 (17)	0.0236 (3)	
H15	0.1550	0.2279	0.3339	0.028*	
C2	0.42134 (11)	0.65916 (14)	0.95732 (18)	0.0307 (4)	
H2A	0.4520	0.6867	0.8847	0.037*	
H2B	0.4563	0.6032	1.0128	0.037*	
C5	0.25158 (10)	0.42072 (13)	0.61192 (17)	0.0256 (3)	
H5	0.1973	0.3967	0.5677	0.031*	
C6	0.32365 (10)	0.37868 (13)	0.56612 (18)	0.0248 (3)	
C9	0.31334 (10)	0.29688 (13)	0.44322 (19)	0.0279 (4)	
H9A	0.3687	0.2854	0.4154	0.034*	
H9B	0.2946	0.2276	0.4764	0.034*	
C13	0.12736 (11)	0.40436 (13)	0.07374 (17)	0.0261 (3)	
H13	0.0869	0.4284	−0.0045	0.031*	
C19	−0.15389 (11)	0.26023 (14)	0.0851 (2)	0.0354 (4)	
H19A	−0.2059	0.2956	0.1020	0.043*	
H19B	−0.1559	0.2587	−0.0185	0.043*	
C8	0.41212 (10)	0.49263 (14)	0.74548 (18)	0.0286 (4)	
H8	0.4665	0.5158	0.7907	0.034*	
C1	0.40403 (13)	0.75029 (16)	1.0549 (2)	0.0393 (4)	
H1A	0.3717	0.8067	0.9983	0.059*	
H1B	0.4576	0.7797	1.1048	0.059*	
H1C	0.3717	0.7227	1.1239	0.059*	

### Atomic displacement parameters (Å^2^)

**Table:**

	*U*^11^	*U*^22^	*U*^33^	*U*^12^	*U*^13^	*U*^23^
S1	0.0281 (2)	0.0190 (2)	0.0491 (3)	0.00118 (15)	0.01076 (19)	−0.00376 (17)
S2	0.0267 (2)	0.0175 (2)	0.0379 (2)	−0.00016 (14)	−0.00212 (17)	0.00027 (15)
Cl1	0.0302 (2)	0.0440 (3)	0.0424 (3)	−0.01370 (18)	0.00921 (19)	0.00073 (19)
O1	0.0252 (6)	0.0339 (7)	0.0321 (6)	0.0000 (5)	0.0014 (5)	−0.0065 (5)
C10	0.0250 (8)	0.0209 (8)	0.0282 (8)	0.0021 (6)	0.0064 (6)	−0.0043 (6)
C17	0.0271 (8)	0.0212 (8)	0.0319 (8)	−0.0036 (6)	0.0014 (7)	−0.0030 (7)
C4	0.0200 (8)	0.0317 (9)	0.0297 (8)	0.0017 (6)	0.0049 (6)	0.0024 (7)
C18	0.0287 (8)	0.0277 (8)	0.0353 (9)	−0.0054 (7)	0.0082 (7)	−0.0024 (7)
C12	0.0379 (9)	0.0231 (8)	0.0266 (8)	−0.0037 (7)	0.0107 (7)	0.0002 (7)
C3	0.0270 (8)	0.0241 (8)	0.0244 (7)	0.0006 (6)	0.0023 (6)	0.0028 (6)
C7	0.0209 (8)	0.0313 (9)	0.0362 (9)	0.0046 (7)	0.0034 (7)	−0.0004 (7)
C16	0.0263 (8)	0.0206 (7)	0.0231 (7)	0.0018 (6)	0.0045 (6)	0.0001 (6)
C11	0.0248 (8)	0.0254 (8)	0.0314 (8)	−0.0045 (6)	0.0094 (6)	−0.0057 (7)
C14	0.0259 (8)	0.0190 (7)	0.0242 (7)	0.0016 (6)	0.0067 (6)	−0.0039 (6)
C15	0.0284 (8)	0.0175 (7)	0.0256 (8)	0.0008 (6)	0.0073 (6)	−0.0011 (6)
C2	0.0287 (8)	0.0326 (9)	0.0287 (8)	−0.0036 (7)	0.0000 (7)	−0.0005 (7)
C5	0.0206 (7)	0.0275 (8)	0.0274 (8)	−0.0032 (6)	0.0013 (6)	0.0044 (7)
C6	0.0240 (8)	0.0211 (8)	0.0283 (8)	0.0011 (6)	0.0020 (6)	0.0045 (6)
C9	0.0246 (8)	0.0227 (8)	0.0356 (9)	0.0017 (6)	0.0031 (7)	−0.0005 (7)
C13	0.0326 (8)	0.0229 (8)	0.0225 (7)	0.0011 (7)	0.0045 (6)	0.0000 (6)
C19	0.0244 (8)	0.0271 (9)	0.0548 (11)	0.0011 (7)	0.0072 (8)	−0.0013 (8)
C8	0.0198 (8)	0.0305 (9)	0.0336 (9)	−0.0005 (6)	−0.0002 (7)	−0.0002 (7)
C1	0.0415 (10)	0.0375 (10)	0.0376 (10)	−0.0058 (8)	0.0037 (8)	−0.0065 (8)

### Geometric parameters (Å, °)

**Table:**

S1—C16	1.8202 (16)	C7—C6	1.388 (2)
S1—C19	1.8209 (18)	C7—C8	1.388 (2)
S2—C17	1.8146 (16)	C7—H7	0.9300
S2—C16	1.8165 (16)	C16—C14	1.510 (2)
Cl1—C11	1.7494 (16)	C16—H16	0.9800
O1—C3	1.371 (2)	C14—C15	1.391 (2)
O1—C2	1.431 (2)	C14—C13	1.396 (2)
C10—C11	1.389 (2)	C15—H15	0.9300
C10—C15	1.396 (2)	C2—C1	1.503 (3)
C10—C9	1.515 (2)	C2—H2A	0.9700
C17—C18	1.515 (2)	C2—H2B	0.9700
C17—H17A	0.9700	C5—C6	1.391 (2)
C17—H17B	0.9700	C5—H5	0.9300
C4—C5	1.379 (2)	C6—C9	1.516 (2)
C4—C3	1.395 (2)	C9—H9A	0.9700
C4—H4	0.9300	C9—H9B	0.9700
C18—C19	1.520 (2)	C13—H13	0.9300
C18—H18A	0.9700	C19—H19A	0.9700
C18—H18B	0.9700	C19—H19B	0.9700
C12—C13	1.383 (2)	C8—H8	0.9300
C12—C11	1.385 (2)	C1—H1A	0.9600
C12—H12	0.9300	C1—H1B	0.9600
C3—C8	1.385 (2)	C1—H1C	0.9600
			
C16—S1—C19	98.37 (8)	C13—C14—C16	118.54 (14)
C17—S2—C16	99.46 (7)	C14—C15—C10	122.22 (15)
C3—O1—C2	118.16 (13)	C14—C15—H15	118.9
C11—C10—C15	116.81 (15)	C10—C15—H15	118.9
C11—C10—C9	122.53 (14)	O1—C2—C1	107.64 (14)
C15—C10—C9	120.65 (14)	O1—C2—H2A	110.2
C18—C17—S2	114.06 (11)	C1—C2—H2A	110.2
C18—C17—H17A	108.7	O1—C2—H2B	110.2
S2—C17—H17A	108.7	C1—C2—H2B	110.2
C18—C17—H17B	108.7	H2A—C2—H2B	108.5
S2—C17—H17B	108.7	C4—C5—C6	121.76 (14)
H17A—C17—H17B	107.6	C4—C5—H5	119.1
C5—C4—C3	119.87 (15)	C6—C5—H5	119.1
C5—C4—H4	120.1	C7—C6—C5	117.46 (15)
C3—C4—H4	120.1	C7—C6—C9	122.32 (15)
C17—C18—C19	113.19 (14)	C5—C6—C9	120.21 (14)
C17—C18—H18A	108.9	C10—C9—C6	112.37 (13)
C19—C18—H18A	108.9	C10—C9—H9A	109.1
C17—C18—H18B	108.9	C6—C9—H9A	109.1
C19—C18—H18B	108.9	C10—C9—H9B	109.1
H18A—C18—H18B	107.8	C6—C9—H9B	109.1
C13—C12—C11	119.27 (15)	H9A—C9—H9B	107.9
C13—C12—H12	120.4	C12—C13—C14	120.33 (15)
C11—C12—H12	120.4	C12—C13—H13	119.8
O1—C3—C8	124.69 (14)	C14—C13—H13	119.8
O1—C3—C4	116.00 (14)	C18—C19—S1	113.71 (13)
C8—C3—C4	119.31 (15)	C18—C19—H19A	108.8
C6—C7—C8	121.77 (15)	S1—C19—H19A	108.8
C6—C7—H7	119.1	C18—C19—H19B	108.8
C8—C7—H7	119.1	S1—C19—H19B	108.8
C14—C16—S2	111.22 (10)	H19A—C19—H19B	107.7
C14—C16—S1	109.25 (10)	C3—C8—C7	119.78 (15)
S2—C16—S1	112.24 (8)	C3—C8—H8	120.1
C14—C16—H16	108.0	C7—C8—H8	120.1
S2—C16—H16	108.0	C2—C1—H1A	109.5
S1—C16—H16	108.0	C2—C1—H1B	109.5
C12—C11—C10	122.54 (15)	H1A—C1—H1B	109.5
C12—C11—Cl1	117.90 (13)	C2—C1—H1C	109.5
C10—C11—Cl1	119.56 (13)	H1A—C1—H1C	109.5
C15—C14—C13	118.78 (15)	H1B—C1—H1C	109.5
C15—C14—C16	122.67 (14)		
			
C16—S2—C17—C18	−59.13 (14)	C16—C14—C15—C10	176.73 (14)
S2—C17—C18—C19	65.33 (18)	C11—C10—C15—C14	0.8 (2)
C2—O1—C3—C8	1.2 (2)	C9—C10—C15—C14	−179.94 (14)
C2—O1—C3—C4	−177.84 (14)	C3—O1—C2—C1	175.34 (14)
C5—C4—C3—O1	176.93 (14)	C3—C4—C5—C6	0.8 (2)
C5—C4—C3—C8	−2.2 (2)	C8—C7—C6—C5	−1.1 (2)
C17—S2—C16—C14	−176.03 (11)	C8—C7—C6—C9	178.18 (15)
C17—S2—C16—S1	61.23 (10)	C4—C5—C6—C7	0.8 (2)
C19—S1—C16—C14	174.28 (11)	C4—C5—C6—C9	−178.52 (15)
C19—S1—C16—S2	−61.87 (10)	C11—C10—C9—C6	71.3 (2)
C13—C12—C11—C10	−1.0 (3)	C15—C10—C9—C6	−107.93 (17)
C13—C12—C11—Cl1	178.80 (12)	C7—C6—C9—C10	−129.56 (17)
C15—C10—C11—C12	0.8 (2)	C5—C6—C9—C10	49.7 (2)
C9—C10—C11—C12	−178.46 (15)	C11—C12—C13—C14	−0.5 (2)
C15—C10—C11—Cl1	−178.94 (12)	C15—C14—C13—C12	2.0 (2)
C9—C10—C11—Cl1	1.8 (2)	C16—C14—C13—C12	−176.95 (15)
S2—C16—C14—C15	−22.01 (19)	C17—C18—C19—S1	−66.73 (18)
S1—C16—C14—C15	102.44 (15)	C16—S1—C19—C18	61.15 (15)
S2—C16—C14—C13	156.87 (12)	O1—C3—C8—C7	−177.15 (15)
S1—C16—C14—C13	−78.68 (16)	C4—C3—C8—C7	1.8 (3)
C13—C14—C15—C10	−2.1 (2)	C6—C7—C8—C3	−0.2 (3)

### Hydrogen-bond geometry (Å, °)

**Table:**

*D*—H···*A*	*D*—H	H···*A*	*D*···*A*	*D*—H···*A*
C19—H19A···O1^i^	0.97	2.40	3.367 (2)	173

Symmetry codes: (i) −*x*, −*y*+1, −*z*+1.
